# The chemokine CXCL1/growth related oncogene increases sodium currents and neuronal excitability in small diameter sensory neurons

**DOI:** 10.1186/1744-8069-4-38

**Published:** 2008-09-24

**Authors:** Jun-Gang Wang, Judith A Strong, Wenrui Xie, Rui-Hua Yang, Dennis E Coyle, Dayna M Wick, Ericka D Dorsey, Jun-Ming Zhang

**Affiliations:** 1 Department of Anesthesiology, University of Cincinnati College of Medicine, Cincinnati, OH 45267-0531, USA

## Abstract

**Background:**

Altered Na^+ ^channel expression, enhanced excitability, and spontaneous activity occur in nerve-injury and inflammatory models of pathological pain, through poorly understood mechanisms. The cytokine GRO/KC (growth related oncogene; CXCL1) shows strong, rapid upregulation in dorsal root ganglion in both nerve injury and inflammatory models. Neurons and glia express its receptor (CXCR2). CXCL1 has well-known effects on immune cells, but little is known about its direct effects on neurons.

**Results:**

We report that GRO/KC incubation (1.5 nM, overnight) caused marked upregulation of Na^+ ^currents in acutely isolated small diameter rat (adult) sensory neurons in vitro. In both IB4-positive and IB4-negative sensory neurons, TTX-resistant and TTX-sensitive currents increased 2- to 4 fold, without altered voltage dependence or kinetic changes. These effects required long exposures, and were completely blocked by co-incubation with protein synthesis inhibitor cycloheximide. Amplification of cDNA from the neuronal cultures showed that 3 Na channel isoforms were predominant both before and after GRO/KC treatment (Na_v _1.1, 1.7, and 1.8). TTX-sensitive isoforms 1.1 and 1.7 significantly increased 2 – 3 fold after GRO/KC incubation, while 1.8 showed a trend towards increased expression. Current clamp experiments showed that GRO/KC caused a marked increase in excitability, including resting potential depolarization, decreased rheobase, and lower action potential threshold. Neurons acquired a striking ability to fire repetitively; IB4-positive cells also showed marked broadening of action potentials. Immunohistochemical labelling confirmed that the CXCR2 receptor was present in most neurons both in dissociated cells and in DRG sections, as previously shown for neurons in the CNS.

**Conclusion:**

Many studies on the role of chemokines in pain conditions have focused on their rapid and indirect effects on neurons, via release of inflammatory mediators from immune and glial cells. Our study suggests that GRO/KC may also have important pro-nociceptive effects via its direct actions on sensory neurons, and may induce long-term changes that involve protein synthesis.

## Background

Altered sodium channel expression in sensory neurons contributes to enhanced excitability and abnormal spontaneous activity after nerve injury or inflammation [1]. However, little is known about mechanisms of this sodium channel regulation. Recent studies suggest important roles for cytokines and chemokines in the nervous system, in both neuropathic and inflammatory chronic pain. They may regulate pain via direct effects on neurons or glia, or via local release of modulators (e.g., prostaglandins; bradykinin) from immune cells [2-6].

We recently described a rat pain model in which the sensory neurons of the L4 and/or L5 dorsal root ganglia (DRG) are inflamed by deposition of zymosan, which stimulates a robust localized inflammatory response without overt nerve injury. This caused abnormal spontaneous activity, along with markedly increased sodium currents in small diameter, primarily nociceptive DRG neurons, an overall increase in excitability [7], and a very striking early increase (15-fold after one day) in the chemokine GRO/KC (growth-related oncogene/keratine-derived chemokine; CXCL1) [8]. We observed similar large increases of GRO/KC in the spinal nerve ligation model [9].

GRO/KC is best known for its role in neutrophil chemotaxis and degranulation early during inflammation. In this regard its effects are similar to those of other CXC family cytokines with an ELF motif, including interleukin-8 (IL-8; CXCL8) in humans [10]. GRO/KC may also have direct roles in the nervous system, including roles in pathological pain. Both GRO/KC and its primary receptor, CXCR2 (IL-8Rb) are expressed in neurons and other cells in the central nervous system, under normal and pathological conditions [11-17]. Less is known about GRO/KC in the peripheral nervous system. However, GRO/KC stimulates calcium influx [18], and release of the pain-related peptide calcitonin gene-related peptide (CGRP) [19] from cultured neonatal DRG neurons. Levels of GRO/KC in inflamed muscle tissue are well correlated with nociceptive behavior [20]. Overall, these studies in peripheral nervous system suggest a pro-nociceptive role for GRO/KC (however, see [21]).

In view of the striking increases in GRO/KC observed in DRG in several pain models, the evidence that this chemokine may play important roles in pathological pain, and the relative dearth of information about its mechanisms of action in pain processing pathways (especially in the periphery), we initiated this study to examine effects of GRO/KC on the properties of small diameter DRG neurons. In light of our previous study showing marked increases in Na^+ ^currents and excitability following local inflammation of the DRG [7], we focused on possible regulation of Na^+ ^currents by GRO/KC in small diameter rat DRG neurons in acute primary culture. Here we report that overnight incubation with GRO/KC, but not acute application, leads to increased excitability, 2- to 4 fold increases in TTX-resistant and TTX-sensitive Na^+ ^currents without altered voltage dependence or kinetic changes, and increases in mRNA abundance of Na channel isoforms that are already present in control cells. The increase in Na^+ ^currents required protein synthesis. The results suggest that GRO/KC may have important pro-nociceptive effects through direct effects on neurons.

## Methods

### Animals

Young female Sprague-Dawley rats (body weight 100–150 g) were housed one or two per cage under a controlled diurnal cycle of 12 h light and 12 h dark with free access to water and food. The ambient environment was maintained at constant temperature (22 ± 0.5°C) and relative humidity (60–70%). All the surgical procedures and the experimental protocol were approved by the institutional animal care and use committee of the University of Cincinnati (Cincinnati, OH, USA).

### Acute culture of sensory neurons

Rats were anesthetized by intraperitoneal injection of pentobarbital sodium (50 mg/kg). The bilateral L4 and L5 DRGs were isolated and the sheath was carefully removed in ice-cold normal Ringer solution. The connective tissue was digested by exposure to Ca^2+^-free solution containing 1.0% collagenase II (Fisher Scientific) for 30 min at 37°C followed by washout in normal Ringer solution for another 10 min. DRGs were then dissociated by trituration with fire-polished Pasteur pipettes. DRG cells were plated onto poly-D-lysine coated glass coverslips in Medium199 (Sigma, St. Louis, MO, USA) containing 10% heat-inactivated FBS and 1000 U/ml each of penicillin and streptomycin. GRO/KC (rat recombinant, from Cell Sciences, Canton, MA, USA) and/or cycloheximide (Sigma) were added to the medium immediately after plating, as indicated, and the DRG cells were incubated at 37°C (5% CO_2 _balance air) before recording. Recordings were made within 30 hours of plating, and cells were selected for recording based on diameter (< 25 μm) and the absence of processes. Isolectin B4 (IB4) conjugated to fluorescein isothiocyante (FITC) (1 μg/μl; Sigma) was added to the culture medium for 30–60 min prior to start of recording sessions to identify non-peptidergic IB4-postive and peptidergic IB4-negative neurons [22,23]. This procedure has been previously shown not to affect the currents measured [24]. Neurons showing a robust fluorescence signal were classified as IB_4_-positive and those displaying no signal at all, IB_4_-negative. In general, the fluorescence was not examined until after completion of current recordings from each cell; i.e., IB4 binding was not used as a criterion in selecting which cells to record. In each individual experiment, we alternated between recording from control and experimental coverslips throughout the recording period. Every attempt was made to compare control and experimental cells taken from the same cultures, though in some cases it was not possible to obtain data from all four types of cells (experimental and control for both IB4-positive and IB4-negative) in a given experiment, particularly when IB4 status was not determined until the end of the recording. Additional controls for inter-animal variability are described in the Results section. Once it had been well established that the proportion of IB4-positive cells did not change with GRO/KC exposure, IB4 binding was sometimes examined prior to recording in order to ensure that approximately equal numbers of each cell type were recorded in each experiment.

### Electrophysiological recording

After over-night culture (16–30 hours), coverslips were transferred to a recording chamber. Whole cell voltage-clamp recordings of small DRG neurons (diameter 15–25 μm) were conducted at room temperature with an AxoPatch-200B amplifier (Molecular Devices Corp, Union City, CA, USA). Patch pipettes (2.5–4.0 MΩ) were fabricated from borosilicate glass. Data were acquired on a Pentium IV computer with the Clampex 8 program (Molecular Devices Corp). The cell capacitance artifact was cancelled by the nulling circuit of the recording amplifier. Voltage errors were minimized by using ≥ 80% series resistance compensation. The current was filtered at 5 kHz and sampled at 50 kHz. The recording chamber was continuously perfused at room temperature with oxygenated bath solution at a flow rate of 1–2 ml/min.

For the current-clamp study of excitability, we measured the threshold current (rheobase), action potential (AP) threshold, resting membrane potential, AP rising rate, AP falling rate, afterhyperpolarization, and input resistance. Any cells without AP were excluded from the study. Only neurons that had stable membrane potential more negative than -45 mV after obtaining the whole cell configuration were used for further study.

For the voltage-clamp study, total Na^+ ^current was divided into tetrodotoxin-resistant (TTX-R) and TTX-sensitive (TTX-S) currents, using the method previously described [7]. Briefly, in most experiments, the TTX-R currents were isolated from the TTX-S currents by using a holding potential of -50 mV. The peak TTX-R current was measured at each potential. Both TTX-R and TTX-S current were evoked by a series of depolarizations from a holding potential of -80 mV. The amplitude of the TTX-S current in each cell was measured during depolarization to -10 mV from -80 mV, based on the distinct kinetic properties of the two currents. At this voltage, TTX-S current was measured as the difference between the early peak current and the current 5 msec after the beginning of the depolarization, a time window in which the TTX-S current essentially completely inactivates but TTX-R current changes little. In experiments in which TTX-S current evoked from -80 mV was measured at additional voltages, a time window of 11 msec (at -20 mV) or 27 msec (at -30 mV) was used, as determined in our previous studies using TTX to verify the separation of the currents.

### Solutions for recording action potentials

The normal bath solution for recording AP contained (in mM) 130 NaCl, 5 KCl, 2 CaCl_2_, 1 MgCl_2_, 10 HEPES, and 10 glucose. The pH was adjusted to 7.4 with NaOH, and the osmolarity adjusted to ~300–310 mOsm with sucrose. The pipette solution contained (in mM) 140 KCl, 1 CaCl_2_, 2 MgCl_2_, 11 EGTA, 10 HEPES, 10 NaCl and 2 Mg ATP. The pH was adjusted to 7.3 with KOH, and osmolarity to ~290–300 mOsm with sucrose.

### Solutions for recording sodium currents

Sodium currents were measured under a reduced sodium gradient to reduce space clamp problems. The Na^+ ^bath solution contained (in mM) 30 NaOH, 110 TMA-Cl, 5 TEA-Cl, 2 MgCl_2_, 1 CaCl_2_, 0.1 CdCl_2_, 10 HEPES, and 10 glucose. The pH was adjusted to 7.4 with HCl, and the osmolarity was adjusted to ~300–310 mOsm with sucrose. The pipette solution contained (in mM) 140 CsCl, 5 NaOH, 2 MgCl_2_, 1 CaCl_2_, 11 EGTA, 10 HEPES, 2 Mg ATP, and 1 Li GTP. The pH was adjusted to 7.2 with CsOH, and the osmolarity to ~290 – 300 mOsm with sucrose. Voltages were not corrected for liquid junction potentials, which were calculated to be < 10 mV in all cases.

### Immunohistochemical staining of GRO/KC receptor CXCR2 in sectioned DRG tissue

Rats were anesthetized and fixed by perfusing 200–300 ml of Zamboni's fixative (4% paraformaldehyde in 0.1 M phosphate buffer, pH = 7.4) through the left ventricle of the heart. The bilateral L4/L5 DRGs were removed and post-fixed in the perfusion fixative for 2 hours at room temperature. The ganglia were horizontally sectioned with a cryostat at a thickness of 8 μm. Tissue sections were incubated in polyclonal rabbit anti-rat antibody to CXCR2 (Santa Cruz Biotechnology, Santa Cruz, CA) at a dilution of 1:200 overnight at 4°C, followed by reaction with secondary antibody Alexa Fluor 594 goat anti-rabbit IgG (H+L) (1:1000, Invitrogen, Carlsbad, CA) for 1 hour.

### Immunohistochemical staining of GRO/KC receptor CXCR2 in dissociated DRG cells

DRG cells from L4/L5 spinal levels were removed from rats, and neurons were isolated and cultured overnight. The cultured cells were fixed with 4% paraformaldeyhde for 30 min at room temperature, washed in ice cold acetone for 1 min, and permeablized with 1% Triton for 10 min. The cells were then incubated with antibody to CXCR2 (1:100, Santa Cruz Biotechnology) and neuronal marker anti-NeuN (1:100, Milipore, Billerica, MA) for 2 hours followed by incubation in secondary antibody conjugated to Alexa Fluor 594(1:1000, Milipore, Billerica, MA) for 1 hour. After drying, the cells were mounted on coverslips with Vector Hard Set mounting medium (Vector Laboratories Inc., Burlingame, CA, USA). In some experiments, IB4 conjugated to FITC (1 μg/μl), instead of anti-NeuN antibody, was added to the culture medium for 30 min before switching to the primary antibody, to identify IB4-positive and IB4 negative neurons. The immunoreactive products were visualized using a confocal microscope and Slidebook 4.1 imaging acquisition software (Intelligent Imaging Innovations, Denver, CO, USA).

### Western blot analysis of CXCR2 in protein extracted from DRG

Western blot analysis was employed to confirm that the CXCR2 antibody used for immunohistochemistry recognized protein in the DRG of the size expected for CXCR2. Bilateral L4/L5 DRGs were isolated and homogenized in RIPA lysis buffer (0.5 M Tris-HCl, pH 7.4, 1.5 M NaCl, 2.5% deoxycholic acid, 10% NP-40, and 10 mM EDTA) (Millipore, Billerica, MA) supplemented just before running the assay with a protease and phosphatase inhibitor cocktail (100 μl of aprotinin, leupeptin, and pepstatin; 500 μl of PMSF, Na_3_VO4, and NaF) (Pierce, Rockford, IL, USA) followed by centrifugation at 4 °C for 40 min. Samples (15 μg of total protein per lane) were subjected to sodium dodecyl sulfate polyacrylamide gel electrophoresis (SDS-PAGE) running in Tris-HEPES-SDS buffer (Pierce, Rockford, IL) followed by electrophoretic transfer to nitrocellulose membrane (Bio-Rad, Hercules, CA, USA) in tris-glycine-SDS transfer buffer. Nonspecific binding sites were blocked with 3% nonfat dry milk (Bio-Rad) in Tween (0.1%)-phosphate-buffered saline (PBST) at room temperature for 1 hour. The CXCR2 antibody (1:300, Santa Cruz Biotechnology) was applied to the blot followed by incubation with immunopure peroxidase conjugated goat anti-rabbit IgG (H+L) (1:20,000, Pierce, Rockford, IL). The blot was developed by exposure to blue-light-sensitive Hyperfilm ECL (GE Healthcare Bio-Sciences Corp) using the enhanced Chemiluninescent (ECL) Western Blotting Detection System (GE Healthcare Bio-Sciences Corp., Piscataway, NJ).

### Quantitative analysis of sodium channel subtype expression

Total RNA was isolated from DRG cultures under conditions similar to those used in electrophysiological recordings, using a Stratagene Absolutely RNA Miniprep Kit (Stratagene, La Jolla, CA, USA). Immediately after isolation, RNA was transcribed into cDNA using an iScript cDNA kit (Bio-Rad). Cells treated overnight with 1.5 nM GRO/KC were compared with control cells from a sister culture. Relative channel expression was determined by quantitative PCR using a Stratagene MX-Pro 3005P. cDNA samples were amplified in triplicate (10 min at 95°C, followed by 40 cycles of 30 sec 95°C, 60 sec 60°C, then 60 sec 72°C with fluorescence measured at the end) in a 25 μl reaction volume (400 nM each forward and reverse primers, SYBR green reaction mix (Roche Applied Science, Indianapolis, IN)). Fluorescence was normalized with the reference dye ROX, and gene expression was normalized to the housekeeping gene hypoxanthine ribosyltransferase (HPRT; [25]), run on the same plate. Primers (Table 1) were selected using the Primer3 program [26] and were designed to include exon-intron boundaries to minimize contamination by genomic DNA. Care was also taken to avoid regions of homology with other Na channels, and primers were examined with an in silico virtual PCR program to ensure that no other products were predicted from rat cDNA or genomic DNA . PCR products were verified by melting point analysis at the end of each experiment and, during protocol development, by gel electrophoresis. Template from control cells was run side-by-side with template from GRO/KC-treated cells from the same culture. The efficiencies of the amplification reactions were estimated directly from the amplification plots using the DART program and a correction for efficiencies was included in the relative expression calculations [27]. Efficiencies were generally in the range of 95 to 103% and were similar for control and GRO/KC treated samples, and across experiments. Hence a single efficiency value was used for control and GRO/KC samples for each gene in any given experiment. Efficiencies were set to 100% for genes with low expression that amplified only during the last few cycles and did not have enough data points for the DART efficiency measurement; low-expressing genes were not studied in quantitative detail. With the exception of the efficiency estimate, amplification data were analyzed using MxPRO software (Stratagene) to determine values for amplification threshold and relative expression.

**Table 1:** Primers used for qPCR experiments:

Name	Rat gene symbol	Rat gene ID	Rat mRNA reference	Primers Forward, reverse	Predicted size (bp)	Position in mRNA
Na_v _1.1	Scn1a	81574	NM_030875.1	atccgagtccgaagatagca	137	1817..1953
				gtctcggggaaaacagtgag		
Na_v _1.2	Scn2a1	24766	NM_012647.1	cggagagttcgtcagtagcc	229	1661..1889
				aagagagactggtgcggaga		
Na_v _1.3	Scn3a	81574	NM_013119.1	aacttggtgccatcaaatcc	182	4513..4334
				cagattcacacccatgatgc		
Na_v _1.4	Scn4a	25722	NM_013178.1	caccgctcttctctgcttct	282	23..304
				gtgggtgtcctggtcttgtt		
Na_v _1.5	Scn5a	25665	NM_013125.1	ttctagctcgaggcttctgc	132	727..858
				tgcgtaaggctgagacattg		
Na_v _1.6	Scn8a	29710	NM_019266.2	tacagtggctacagcggcta	274	2008..2281
				tgtttgtgaccacgctcatt		
Na_v _1.7	Scn9a	78956	NM_133289	gacagcttcttccagaggtgataata	215	1922..2024
				ccatggtggacatttttgtct		
Na_v _1.8	Scn10a	29571	XM_001078257	cacggatgacaacaggtcac	151	1610..1760
				gatcccgtcaggaaatgaga		
Na_v _1.9	Scn11a	29701	NM_019265	ggcagccaagtcaatctttc	233	679..911
				gggccacagttgtgcttaat		
HPRT	Hprt	24465	NM_012583	gcagactttgctttccttgg	278	495..772
				tactggccacatcaacagga		

### Data analysis

**Figure 1:**
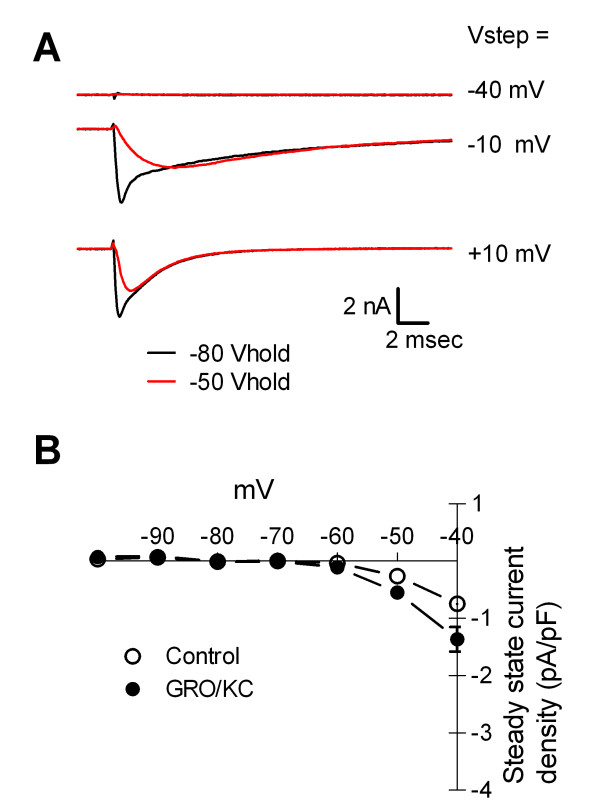
**GRO/KC incubated cells have Na^+ ^currents that are qualitatively similar to those seen in control cells**. A. Examples of current traces elicited from holding potential of -80 mV (darker, smaller traces) or -50 mV. As in control cells, the currents elicited from -80 mV have a faster component, which is TTX-sensitive (see text). B. Persistent Na^+ ^currents activating at -60 to -50 mV were not observed. Average leak-subtracted steady state current is plotted against the step potential. Holding potential was -80 mV.

Data were analyzed using Clampfit 9 (Molecular Devices Corp), Graphpad Prism (GraphPad Software, Inc., San Diego, CA, USA), and Origin 7 (Origin Lab Corp., Northampton, MA, USA). Currents were normalized by cell capacitance. The ohmic portion of the observed peak Na^+ ^currents was measured from current steps to -100 through -60 mV and subtracted. Data are expressed as mean ± standard error of the mean (SEM). Differences in proportion of cells binding IB4 were examined with Fisher's exact test. Statistical significance of differences between average values in experimental and control neurons were analyzed by Student's t-test or, for data that were not normally distributed, the Mann-Whitney rank sum test, using SigmaStat Software (Systat Software, Inc., San Jose, CA, USA). In the case of multiple comparisons over a voltage range for activation data, the data were analyzed by two-way repeated measures ANOVA (RM ANOVA), with pairwise multiple comparison (Holm-Sidak method) to determine at which voltages the differences between experimental and control cells were significant if an overall effect of GRO/KC treatment was observed. ANOVA (or Kruskal-Wallis test on ranks, for data not showing normal distribution) was used to analyze experiments with more than two experimental groups. Activation data was fitted to the Boltzmann equation (Figure 1) was done using Graphpad Prism software. Significance of fold-changes in relative Na channel expression in qPCR experiments was done using a ratio t-test. Significance was ascribed for p < 0.05.

## Results

### Incubation with GRO/KC enhances Na^+ ^current density without affecting other parameters

Small diameter DRG neurons were acutely cultured and incubated overnight with 1.5 nM rat (recombinant) GRO/KC or vehicle. The Na^+ ^currents were measured in small diameter neurons with whole cell patch clamp techniques. After incubation in GRO/KC, voltage-activated Na^+ ^currents had overall properties (except for amplitude) similar to those we have previously described in cells cultured from control and locally inflamed DRG [7]. The Na^+ ^currents in both control and GRO/KC treated cells activated at -30 to -20 mV (Figure 1). Persistent Na^+ ^currents attributed in previous studies to the Nav 1.9 isoform, which activate at potentials near -60 mV under recording conditions similar to ours [28,29], were observed in neither control nor GRO/KC treated cells. We observed small sustained inward currents -40 mV in less than 10% of cells, and never observed inward currents at potentials of -50 or -60 mV (Figure 1; compare to [28,29]). Na^+ ^currents from GRO/KC treated cells (like those previously observed in control cells and inflamed cells) had a faster component that was blocked by 50 nM tetrodotoxin (TTX; n = 6 GRO/KC treated cells; data not shown) or by using a holding potential of -50 mV (Figure 1 and [7]). TTX-resistant currents, which have slower kinetics, could be isolated by using a holding potential of -50 mV which inactivated TTX-sensitive currents.

**Figure 2:**
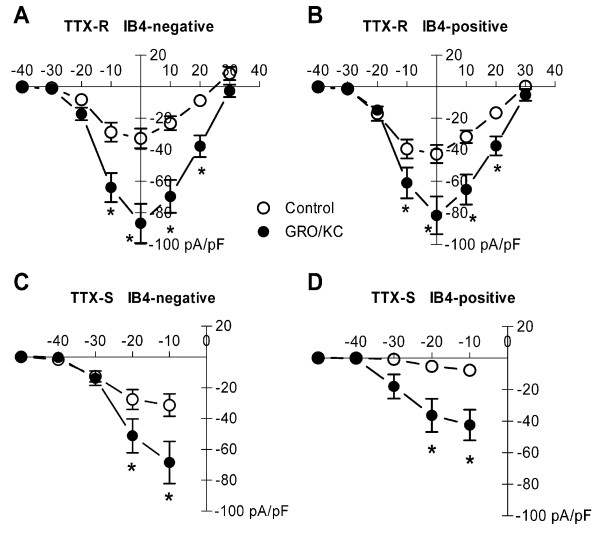
**Overnight incubation with GRO/KC (1.5 nM) increases Na^+ ^current density**. A. TTX-R current density significantly increased in IB4-negative cells after GRO/KC incubation. P < 0.0001 for overall effect. Individual voltages at which the difference between control and GRO/KC treated cells was significant are indicated by the * symbol (two-way RM ANOVA with Holm-Sidak method for Pairwise Multiple Comparisons). B. A similar but smaller enhancement of TTX-R current was observed in IB4-positive cells (overall p value = 0.0016). TTX-S current density measured at -30 to -10 mV as described in methods, also significantly increased after GRO/KC incubation in both IB4-negative (C; and IB4-positive cells (D). Data are from 9 cultures; N = 53 control cells and 54 GRO/KC treated cells.

As shown in Figure 2, GRO/KC incubation (16 to 30 hours) significantly increased the density both TTX-R and TTX-S currents. These effects were seen in both IB4-positive and IB4-negative neurons. These results are from 53 control cells (26 were IB4-positive) and 54 GRO/KC treated cells (28 were IB4-positive). In order to ensure that this primary finding was not confounded by inter-animal variability, we also analyzed the subset of data in which currents measured in control cells could be compared with that measured in GRO/KC treated cells from the same batch (animal), in side-by-side experiments. Statistical analysis similar to that shown in Figure 1 was then conducted using the batch averages (n = 4) instead of the individual values for each cell. Despite the much smaller N, the increase in TTX-R current was still statistically significant in both IB4-positive (overall p = 0.019) and IB4-negative (p = 0.002) cells. The GRO/KC induced increases in TTX-S currents showed the same trends as shown in Figure 2, though these did not reach significance with the smaller N value when batch averages were analyzed (p = 0.1 for IB4-negative cells and p = 0.2 for IB4-positive cells). The proportion of IB4-positive cells was not significantly altered by GRO/KC treatment (p = 0.8, Fisher's exact test).

The higher (2 – 3 fold) TTX-S current density observed in IB4-negative compared to IB4-positive cells was significant in both control cells (p = 0.03, Mann-Whitney test) and GRO/KC treated cells (p = 0.02, Mann-Whitney test). There was a tendency for the peak TTX-R current (at 0 mV) to be higher in IB4-negative cells which however did not reach significance in either control cells (28% higher, p = 0.16, Mann-Whitney test) or in GRO/KC treated cells (32% higher, p = 0.19, t-test).

**Figure 3:**
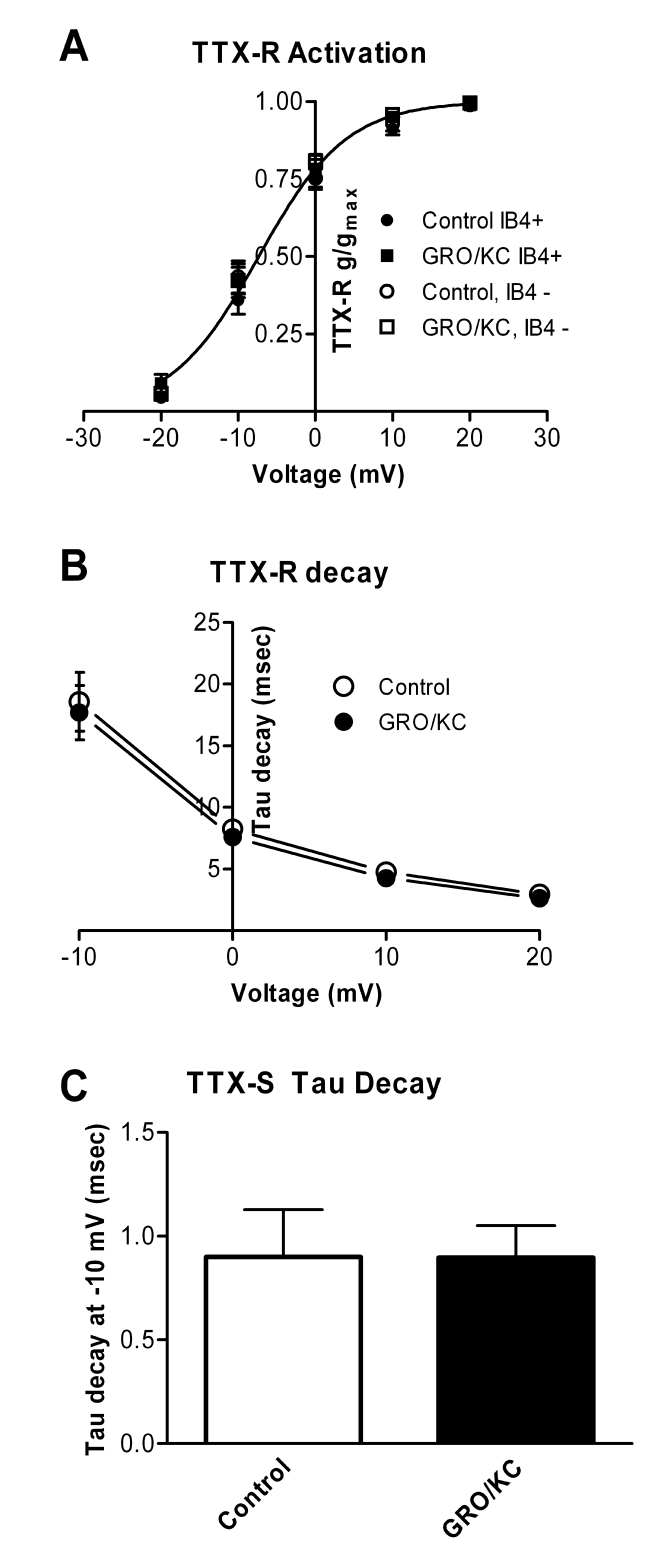
**Overnight incubation with GRO/KC does not affect decay time constants or activation of Na^+ ^currents**. A. Activation data for the TTX-R current was fit by a standard Boltzmann equation. No differences were found in the fit parameters between the 4 experimental groups (p = 0.22). The plotted line represents the best fit to all the data, and has a V_1/2 _value of -7.3 mV and a slope factor of 5.7. B. Time constants for the decay of TTX-R current were obtained by fitting single exponentials to the falling phase of currents evoked from a holding potential of -50 mV. No significant difference between the two groups was observed (two-way RM ANOVA). C. Time constants for the TTX-S current at were obtained by fitting the decaying phase of the current with the sum of two exponentials. The slower of these corresponded to the time constant observed in the TTX-R current, and the faster time constant was used as the value of the TTX-S decay time constant. No significant difference between the two groups was observed (Mann-Whitney test, p = 0.18). Data from IB4-positive and IB4-negative cells were combined as no difference between these groups in decay time constants was observed.

The voltage dependence of activation for the TTX-resistant current was not significantly affected by GRO/KC incubation (Figure 3A) and was not different between IB4-positive and IB4-negative cells. The activation curve for the combined data was well fit by a Boltzmann equation with V_1/2 _= -7.3 mV and a slope factor of 5.7. The time course of decay for the TTX-R current during depolarizing pulses was also unaffected by GRO/KC (Figure 3B). Similarly, the time constant for decay of the TTX-S current at -10 mV was not affected by GRO/KC incubation (Figure 3C). The TTX-sensitive current became too fast to accurately voltage clamp at more positive potentials, so its activation curve was not determined in these experiments. However, the TTX-sensitive current observed during steps from -80 to -10 mV or below, where it is readily kinetically separated from the TTX-resistant current (see Methods), showed a qualitatively similar threshold for activation after GRO/KC incubation (see Figure 2C and 2D).

**Figure 4:**
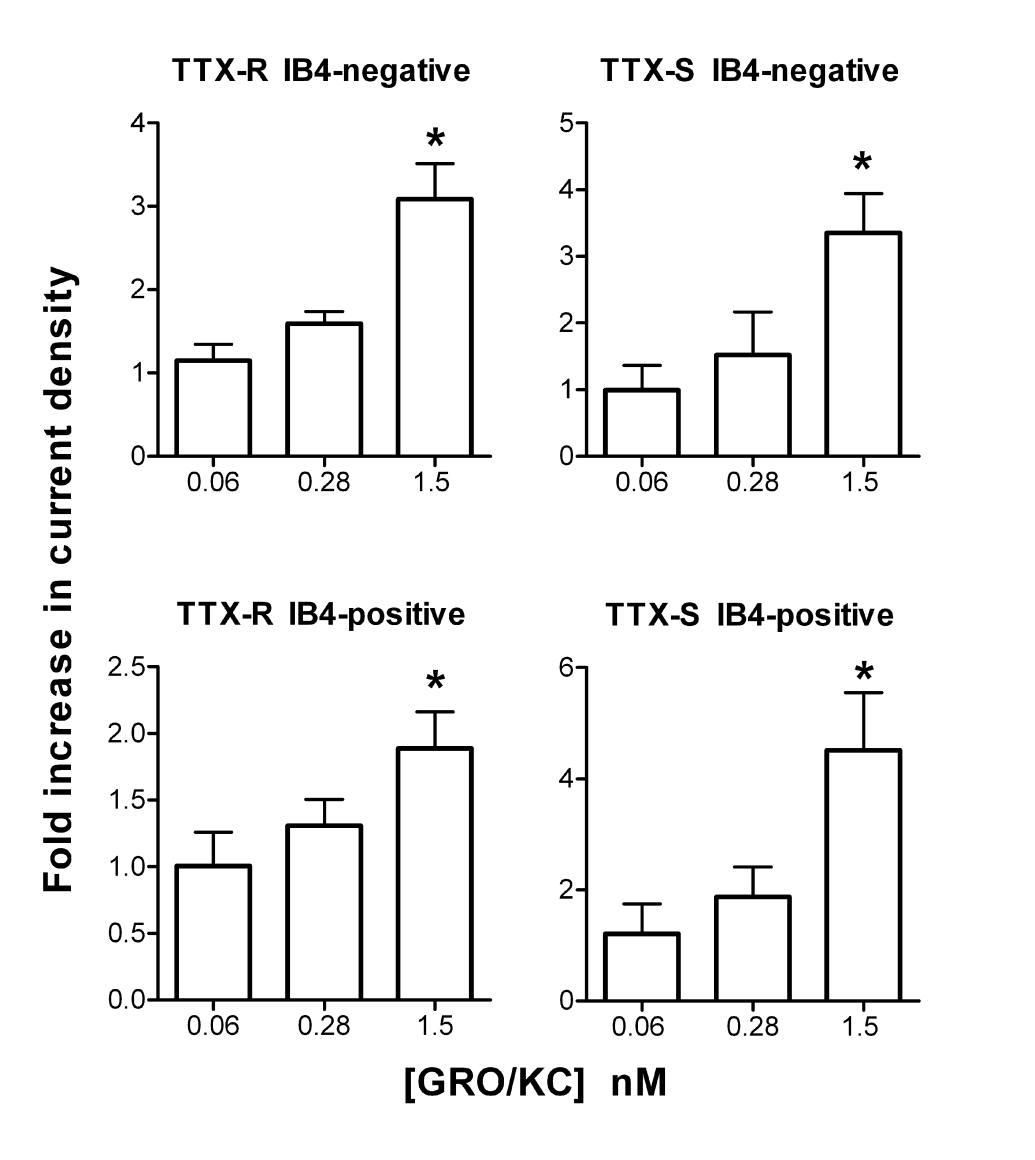
**Lower doses of GRO/KC do not increase Na^+ ^current density**. Current densities were measured as in Figure 1, after overnight exposure to the indicated concentration of GRO/KC. For each concentration tested, data have been normalized to the control values obtained in cells from the same cultures with no GRO/KC treatment. *, significantly different from control. TTX-R current at 0 mV was used for this analysis. N = 27 cells for 0.06 nM dose (plus 17 control cells from the same cultures); 10 cells for 0.28 nM dose (plus 14 control cells), and 54 cells for 1.5 nM dose (plus 53 control cells).

Lower doses of GRO/KC were also tested in similar experiments. Figure 4 shows the Na^+ ^current density measured at two lower doses of GRO/KC, 0.28 and 0.06 nM. As shown, only the highest dose tested, 1.5 nM, gave a significant increase over control values, In Figure 4, data from control cells were obtained for the experiments at each separate dose. However, similar results were obtained if the data were analyzed by comparing current densities at each individual dose to the combined control values.

### Enhancement of Na^+ ^currents by GRO/KC is blocked by protein synthesis inhibitor

**Figure 5:**
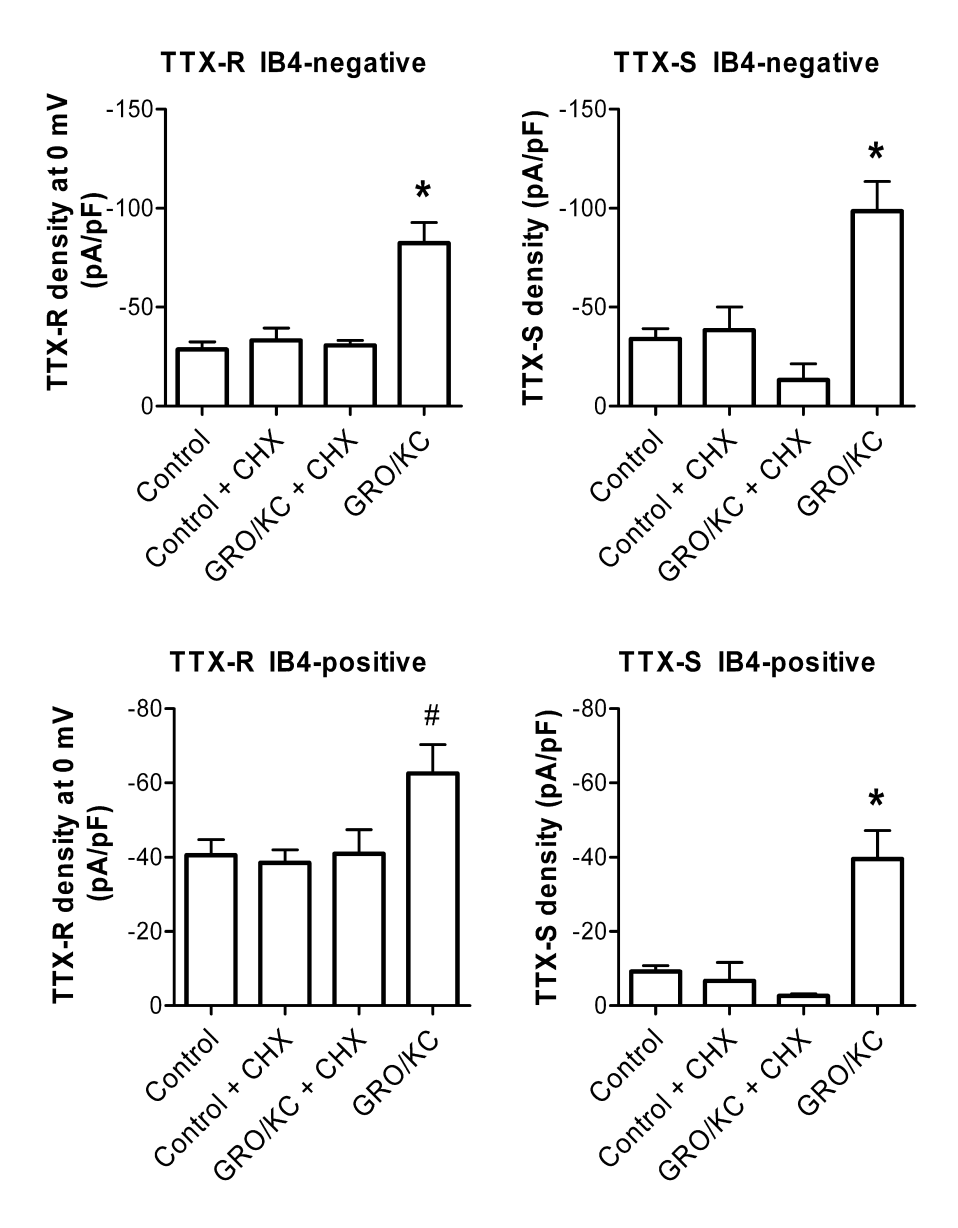
**Effect of the protein synthesis inhibitor cycloheximide (CHX) on enhancement of Na^+ ^currents by GRO/KC incubation**. Cycloheximide (3.5 μM) or vehicle (DMSO) was added to cell cultures just before addition of 1.5 nM GRO/KC or vehicle, and TTX-S and TTX-R Na currents were measured 16 to 30 hours later. *, significantly different from all other groups; #, significantly different from control (one-way ANOVA followed by Tukey's multiple comparison test). Data are from 1 set of experiments (3 cultures) comparing 22 control and 29 CHX treated cells, and a second set of experiments (2 cultures) comparing 14 GRO/KC treated cells with 22 GRO/KC + CHX treated cells. Additional data, from Figure 1, are included in the control and GRO/KC data groups. Analysis omitting this additional data gave similar results, except that the TTX-R current in IB4-positive cells showed no significant differences between groups.

We were unable to observe increases in Na^+ ^currents following acute (5 minute) application of 1.5 nM GRO/KC (n = 5 IB4-positive and 7 IB4-negative cells). The finding that GRO/KC enhanced the magnitude of Na^+ ^currents without marked shifts in voltage dependence activation or kinetics (Figure 2, 3), and was observed only after a period of incubation, suggested that the primary effect was an increased number of Na^+ ^channels, and hence that protein synthesis might be required. To test this idea, we conducted experiments using the protein synthesis inhibitor cycloheximide. The results are shown in Figure 5. In general, cycloheximide prevented the GRO/KC-induced increases in Na^+ ^current density without significantly affecting baseline current densities. In both IB4-positive and IB4-negative cells, only the GRO/KC without CHX group differed significantly, from all three other groups.

### The Na channel expression profile indicates upregulation of previously expressed channel types

**Figure 6:**
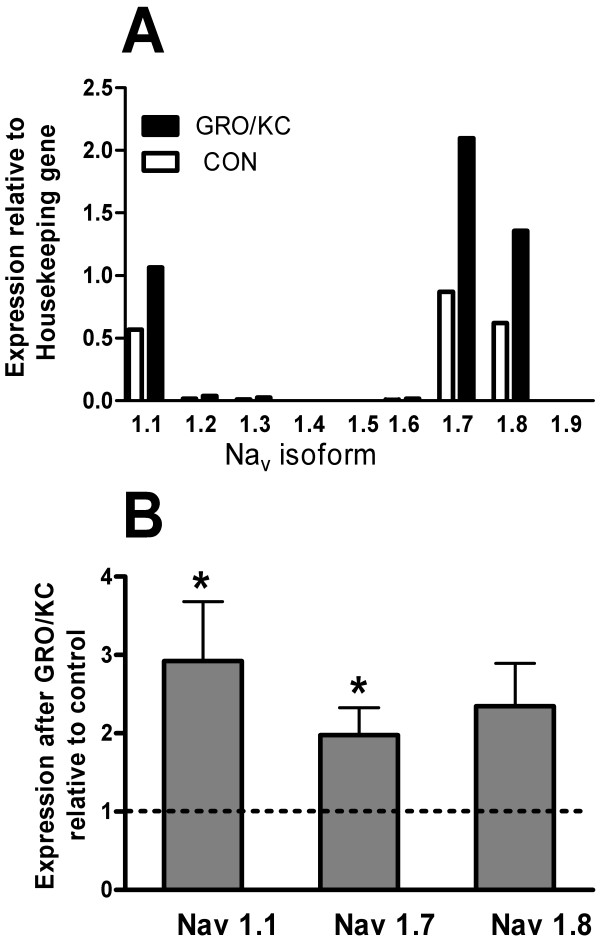
**Expression of Na channels Na_v_1.1 through Na_v _1.9 in cultured DRG cells before and after GRO/KC incubation**. cDNA template was reverse-transcribed from RNA that had been isolated from control cells and cells from the sister cultures treated overnight with GRO/KC (1.5 nM). Expression of each gene was normalized to that of the housekeeping gene HPRT in the same batch of cDNA, determined during the same PCR amplification. A, profile of Na channel expression relative to HPRT. B, fold-change in expression of the 3 highly expressed Na channels after GRO/KC treatment. *, significantly different from control (= 1.0, dotted line), ratio t-test. Values shown are averages from 4 separate cultures.

Nine different genes for voltage-gated Na channels have been described, with Na_v_1.1 – Na_v_1.7 mediating TTX-sensitive current and Na_v _1.8 and 1.9 mediating TTX-resistant currents [30]. A simple explanation for the observed effects of GRO/KC on Na channel currents would be enhanced expression of channels with properties similar to those seen in control cells. As an initial test of this possibility, we used quantitative PCR methods to quantify relative changes in the amounts of Na channel mRNA present in DRG neurons after overnight incubation in 1.5 nM GRO/KC. Culture conditions were kept as close as possible to those used for the electrophysiological experiments. Expression data were normalized to that of the housekeeping gene HPRT. Of the nine Na channels, 3 showed much higher expression relative to HPRT than the others: Na_v _1.1, 1.7, and 1.8 (Figure 6A). Na_v _1.4 (a form found primarily in skeletal muscle), and 1.5 (a form found primarily in cardiac muscle), were undetectable or showed product only during the final few cycles. Na_v _1.9 (which is thought to mediate persistent Na currents in nociceptors) was also very low abundance, with relative expression on the order of 10^-6 ^times that of Nav 1.8. In contrast, in mRNA isolated from a whole DRG, we observed Na_v_1.9 mRNA with an abundance similar to that of Na_v _1.8. This profile of expressed channels was grossly similar both before and after GRO/KC treatment; no previously low abundance channels were markedly upregulated by GRO/KC. The fold- increases in Na_v _1.1, 1.7, and 1.8 were between 2 and 3 fold, roughly similar to the observed increase in current densities (Figure 6B); this measurement is less sensitive to the efficiency correction. These fold-increases were significant except in the case of Na_v _1.8, which showed a nonsignificant trend to similarly increase (p = 0.08).

### Distinct effects of GRO/KC on the excitability of IB4-positive and IB4-negative neurons

In order to determine the likely physiological effects of GRO/KC, excitability parameters were measured after a 1.5 nM GRO/KC incubation similar to that used to study Na^+ ^currents. As shown in Table 2, the overall effect of GRO/KC treatment was a marked increase in excitability, including a reduced action potential threshold, depolarized resting potential, and decreased rheobase which were observed in both IB4-negative and IB4-positive cells.

**Table 2:** Membrane properties of isolated control and GRO/KC treated neurons in whole cell current clamp experiments

**Cell and AP Properties**	**IB4-negative cells**		**IB4-positive cells**	
	
	**Control**	**GRO/KC**	**Control**	**GRO/KC**
Number of Cells	26	22	25	18
Diameter, μm	20.0 ± 0.8	21.2 ± 0.7	22.8 ± 0.5	20.2 ± 0.9*
Cell Capacitance, pF	17.6 ± 1.3	20.0 ± 1.2	19.0 ± 1.3	16.8 ± 1.7*
Resting Potential, mV	-63.0 ± 1.6	-56.0 ± 1.4**	-64.6 ± 1.4	-55.4 ± 1.9 **
Input Resistance, MΩ	1244.0 ± 144.3	986.1 ± 99.4	1062.0 ± 64.6	1427.4 ± 92.3**
AP Rheobase, pA	60.4 ± 8.4	21.1 ± 2.1**	76.2 ± 14.2	51.1 ± 11.2*
AP Threshold, mV	-19.7 ± 0.9	-28.6 ± 1.4**	-16.6 ± 0.6	-25.4 ± 1.3**
AP Rising Rate, mV/ms	106.8 ± 9.6	112.5 ± 10.7	103.4 ± 8.8	102.4 ± 12.0
AP Falling Rate, mV/ms	49.7 ± 4.8	61.9 ± 7.3	43.4 ± 3.8	39.9 ± 5.0**
AP Amplitude, mV	64.8 ± 1.7	74.8 ± 1.8**	63.1 ± 1.9	71.1 ± 1.5 **
AP Duration, ms	6.6 ± 0.4	7.3 ± 0.5	8.3 ± 0.5	14.0 ± 1.0**
AHP Amplitude, mV	3.5 ± 0.5	5.9 ± 0.6 **	3.0 ± 0.8	4.5 ± 0.7
AHP Duration, ms	10.8 ± 2.4	14.7 ± 0.8*	7.7 ± 1.7	9.9 ± 1.4

Values are mean ± SEM. AP, action potential; AHP, afterhyperpolarization. * P < 0.05, ** P < 0.01, comparing GRO/KC treated vs. Control cells with the same IB4 status (Student's t-test or Mann-Whitney test). Rheobase is defined as the smallest depolarizing current to elicit at least one action potential within 80 msec. Action potential parameters were measured on this first elicited action potential.

**Figure 7:**
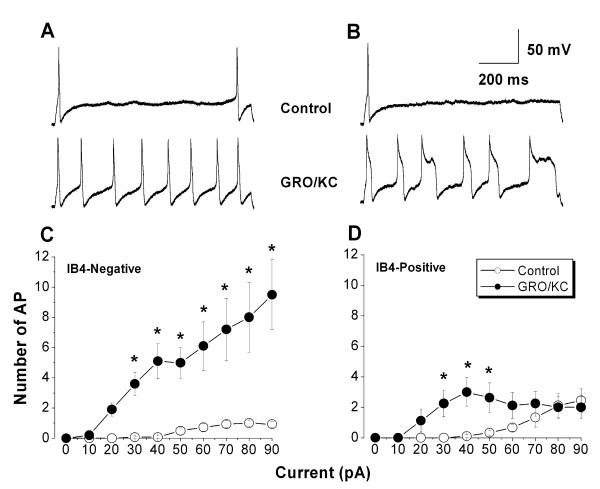
**Small diameter neurons acquire the ability to fire repetitively after GRO/KC incubation**. A, Examples of the voltage response to a 90 pA 1 second current injection in a control IB4-negative neuron (top) and in an IB4-negative neuron incubated overnight in GRO/KC (1.5 nM) (bottom). B. Examples of the voltage response to a 50 pA current injection in control (top) and GRO/KC incubated (bottom) IB4-positive neurons. Same scale as A. C, D: Average number of action potentials during a 1 second current injection as a function of current amplitude in IB4-negative (C) and IB4-positive (D) cells. *, significant difference between GRO/KC and control cells at the indicated current value (two-way RM ANOVA with Holm-Sidak post test). N = 8 to 14 cells per group; data combined from 3 different cultures.

Initially, the experiments shown in Table 2 were conducted by injecting short-duration (80 msec) current pulses, and it was evident that after GRO/KC incubation many cells fired action potentials for the entire duration of these short depolarizing pulses. In order to examine this effect in more detail, subsequent experiments were conducted using much longer (1 s) current pulses. As shown in Figure 7, such long pulses demonstrated a striking ability for repetitive firing that developed after GRO/KC incubation. In IB4-negative cells, multiple action potentials (up to 27) could be observed during the 1 second current injection, and these lasted throughout the entire pulse. IB4-positive cells also acquired the ability to fire throughout the 1 second current injection after GRO/KC incubation. In this case the number of action potentials per pulse did not increase as dramatically; rather, the action potentials tended to become very broad (Figure 7B and Table 2), though still continuing until the end of the 1 second current injection. The significant increase in action potential width (68%) seen in IB4-positive cells in Table 2 may be a conservative estimate, because those measurements are from the first evoked action potential and action potentials tended to broaden even more during the course of a long depolarizing pulse (Figure 7B).

### CXCR2 is expressed in both IB4-positive and IB4-negative neurons

**Figure 8:**
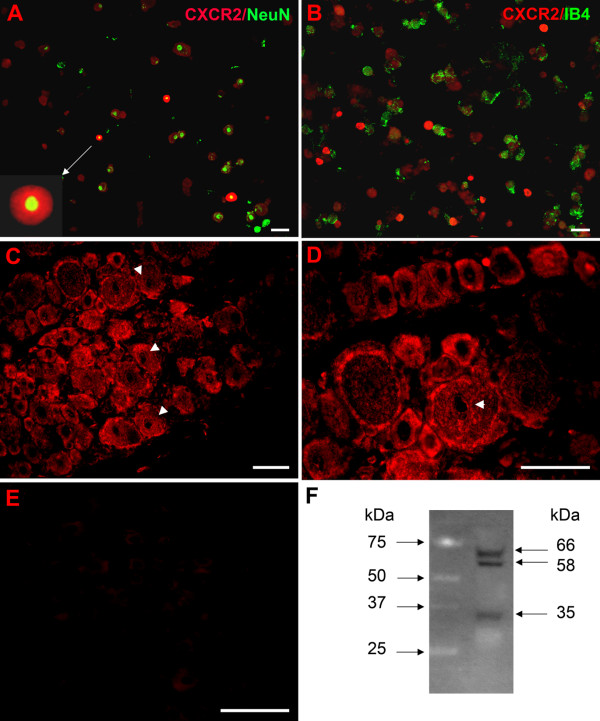
**Immunohistochemical detection and immunofluorescence double labelling of CXCR2 in acutely dissociated DRG neurons and in DRG sections from rat**. A: Double staining of the dissociated DRG cells with anti-CXCR2 and anti-NeuN showing that high CXCR2-expressing cells are neuronal cells, and that the more modest staining levels observed occur in almost all DRG neurons. B: Neurons with intense CXCR2 staining are all IB4-negative. C: CXCR2 staining in DRG sections showing expression on plasma membrane of most neurons, of small, medium and large diameters, with high levels expression in some smaller neurons. CXCR2 is also expressed in the nuclear membrane in some cells (arrows), as can be seen more clearly in the higher magnification view in D. E: Negative control. DRG sections incubated with primary antibody preabsorbed with 30 fold excess of antigen did not show any immunoreactivity. Scale bar = 50 μm. F. Western blot of protein isolated from DRG neurons, using the same antibody (1:300) as a probe. Left panel: standard. Right panel: CXCR2.

Almost all dissociated DRG neurons including IB4-positive and IB4-negative neurons displayed CXCR2 staining (Figure 8A, B). In addition, CXCR2 staining was extremely intense in a subset of DRG cells. The intensely stained cells had an average diameter of 21.9 ± 0.9 μm (n = 3 rats) and were all IB4-negative. However, the number of high CXCR2 expressing cells was very small, approximately 2.4% of all DRG cells, making it likely that the other data reported in this study have a very small contribution from this subset of neurons with extremely high expression levels.

In DRG sections, CXCR2 was expressed in most neurons, whether small, medium, or large diameter (Figure 8C, D). An antibody directed to the C terminus of CXCR2 (supplier: Santa Cruz) displayed intense staining of plasma membrane and, in some cells, the nuclear membrane, with no immunoreactivity in the nucleoli. Although there was no apparent difference in CXCR2 staining of the plasma membrane between most small and large neurons, a subset of small DRG neurons displayed very high density staining, similar to observations made in the dissociated cells. Sections or dissociated cells incubated with primary antibody preabsorbed with 30 fold excess of antigen did not show any immunoreactivity (Figure 8E). In order to further validate the antibody used for the CXCR2 staining, we conducted western blot analysis of protein extracted from whole DRGs. Using the same antibody from Santa Cruz at 1:300 dilution, the western blot showed a strong doublet of 58–66 kDa and a lighter staining band of 35 kDa. An example is shown in Figure 8F; similar results were obtained in 3 replicate experiments.

## Discussion

In this study we found that overnight incubation with GRO/KC (CXCL1), a chemokine that is rapidly upregulated in the DRG in several different models of chronic pain, caused increased excitability and marked upregulation of Na^+ ^current amplitudes in acutely isolated small diameter DRG neurons in short-term culture. This upregulation required protein synthesis. Many studies on the role of chemokines in pain conditions, including GRO/KC (e.g.,[31]), have focused on their indirect effects on neurons, via stimulation of release of inflammatory mediators from immune and glial cells that then act on sympathetic and sensory neurons. Our study suggests that GRO/KC might also have important pro-nociceptive effects via its direct actions on sensory neurons. The majority of small diameter DRG neurons are nociceptors. The striking increase in excitability after GRO/KC incubation, including the ability to fire continuously during long depolarizations, would be expected to profoundly increase the sensitivity to painful stimuli in vivo – both by increasing the sensitivity of nociceptors to stimuli, and by increasing the release of excitatory neurotransmitters in the dorsal horn. It will be important to conduct experiments to interfere with GRO/KC production or action in the DRG in vivo, to confirm the pro-nociceptive roles for this chemokine suggested by this in vitro study.

Our results have some interesting similarities to those reported by Liu et al. [32], who found that chronic (24 h) exposure of cultured small diameter capsaicin-sensitive trigeminal neurons to another pro-inflammatory cytokine, interleukin 1-β (IL-1β), led to a 67% increase in TTX-S (but not the TTX-R) Na^+ ^current, without changes in voltage dependence. As in our study, acute applications did not increase Na^+ ^current (and in fact caused slight decreases in the Na^+ ^current), and the cytokine receptor was shown to be expressed on the neurons.

GRO/KC actions are thought to be mediated by the CXCR2 receptor. As discussed in the Introduction, the presence of this receptor on neurons (including neonatal DRG neurons in vitro) has been previously observed with a variety of methods. The effects reported here are most likely to be due to a direct effect of GRO/KC on the neurons, because: (1) effects were obtained in low density, acute cultures, in which non-neuronal cells were very sparse and intercellular mediators would be diluted by the culture medium; and (2) using an antibody to the CXCR2 receptor, we demonstrated that the receptor was present on the membranes of virtually all of the small diameter neurons in our cultures. Western blot analysis of DRG proteins, using the same antibody, revealed a doublet band at 58–66 kDa and a lighter band of 35 kDa. For comparison, Xiong et al. [33] reported a band of approximately 58 kDa in Western blots of CXCR2 receptor in rat hippocampus. The human receptor, which has an almost identical predicted molecular weight of 40.7 kDa based on amino acid sequence (vs. 40.5 kDa for the rat receptor), has been reported to have an apparent molecular weight of 56 kDa when isolated from neutrophils, due to the presence of 2 N-linked 9 kDa carbohydrate moieties [34]. Interestingly, this same group reported additional forms of the human receptor, of 38 and 40 kDa that were shown to differ in their carbohydrate groups.

The inability of GRO/KC to increase Na^+ ^currents after acute application, or in the presence of cycloheximide, provides evidence that protein synthesis is required for the effect on Na^+ ^currents. The simplest explanation would be that additional Na^+ ^channels are synthesized after GRO/KC incubation, as this would be consistent with the observation that only the magnitudes of the currents are altered, not their activation range or decay time constants. Our measurements of Na channel subtype expression were consistent with this view. However, we cannot entirely rule out other possibilities such as synthesis of proteins that modulate channels already present. The CXCR2 receptor is a G-protein-coupled receptor that has been shown to activate a number of different signalling pathways in neurons, including intracellular Ca^2+^, inositol tris-phosphate, MAP kinases, and CREB (reviewed in [35]). In turn all of these pathways can lead to enhanced transcription and protein synthesis through a number of signal transduction pathways. In addition, some G-protein coupled receptors, including the closely related CXCR4 receptor, have been shown to be present in the nucleus as well as on the plasma membrane, where they are thought to activate nuclear effects through additional pathways (for review, see [36]). This is particularly interesting in light of our observation of nuclear staining for CXCR2 in some cells in addition to plasma membrane staining in DRG neurons. While artifactual nuclear staining can be a problem with immunohistochemical methods, this seems somewhat less likely given the observation that not all nuclei showed staining in our specimens.

We initially identified GRO/KC as a cytokine that might play important roles in pathological pain because it showed marked early increases in a pain model in which localized inflammation of the DRG is induced by zymosan [8]. Using the same model, we found that localized inflammation led to marked increases in TTX-S and TTX-R currents in small diameter DRG neurons, as well as increased excitability, as determined by patch clamp studies in acutely isolated neurons [7]. That study and the present study have certain common findings: both localized inflammation and GRO/KC incubation give striking increases in the magnitude of TTX-S current in both IB4-positive and IB4-negative cells, without obvious changes in inactivation time constant or voltage dependence. Both lead to increased excitability, though these effects are larger for GRO/KC treatment. One difference is that localized inflammation led to increased TTX-R current only in IB4-positive cells, while GRO/KC incubation also increased this current in IB4-negative cells. The comparison of these two studies suggests that GRO/KC elevations in the localized inflammation model provide one plausible explanation for some, but not all of the changes in ion currents and excitability observed in this model. We observed effects on excitability and Na^+ ^currents at a relatively low GRO/KC concentration of 1.5 nM, which is well within the physiological range for most peptide receptor actions. In one study, GRO/KC-stimulated (acute) Ca^2+ ^elevation in isolated DRG neurons could be observed at GRO/KC concentrations of 6.4 nM but was not significant at 1.2 nM [19]. In cells expressing cloned CXCR2 receptors, the binding constant for GRO/KC was 0.2 nM [37]. Thus it seems plausible that the effects reported here could occur in vivo.

The TTX-R currents described here, as in our previous study, had characteristics attributed to the Na_v_1.8 isoform. Our recording conditions would have minimized contributions from the persistent current (thought to be mediated by the Na_v_1.9 isoform) due to slow inactivation at the holding potentials used, and/or because the current may "washout" during whole cell patch clamp [28,38]. Other studies also suggest that the persistent current would be quite small under our recording conditions (adenosine triphosphate in the pipette, Cl^- ^as the anion) [29]. In our qPCR experiments we observed that the Na_v_1.9 isoform was very low abundance under our culture conditions, though it had levels similar to Nav 1.8 in cDNA derived from whole DRG. Hence the lack of Na_v _1.9 currents in our experiments may be related to the acute culture process; some studies of the persistent current used shorter culture times than we used in this study. The TTX-S current in DRG neurons is mediated by a number of different isoforms, which are not readily distinguished electrophysiologically. Some of the excitability changes we observed after GRO/KC incubation, such as resting membrane depolarization and increased input resistance, are not readily explained by the increased Na^+ ^currents observed in the voltage clamp experiments. Hence it is highly likely that additional ion currents can be modulated by GRO/KC. This was also true of localized inflammation, which upregulates voltage activated K^+ ^currents as well as Na^+ ^currents [7].

## Conclusion

In summary, the pro-inflammatory cytokine GRO/KC (CXCL1), which markedly increases in DRG during the early phase of several models of pathological pain, may exert important pro-nociceptive effects in part through its direct actions on nociceptive neurons.

## Competing interests

The authors declare that they have no competing interests.

## Authors' contributions

JW and RY conducted the patch clamp experiments and data analysis. JAS drafted the manuscript and participated in qPCR experiments, data analysis, statistical analysis, and experimental design. WX designed and conducted the immunohistochemical experiments. DEC established the qPCR protocols and participated in data analysis. DMW conducted qPCR experiments and participated in data analysis. EDD participated in qPCR experiments, and obtained and analyzed immunohistochemical data. JZ conceived of the study, and participated in its design and coordination and helped to draft the manuscript. All authors read and approved the final manuscript.
